# Altered diversity and composition of the gut microbiome in patients with cervical cancer

**DOI:** 10.1186/s13568-019-0763-z

**Published:** 2019-03-23

**Authors:** Zhongqiu Wang, Qingxin Wang, Jing Zhao, Linlin Gong, Yan Zhang, Xia Wang, Zhiyong Yuan

**Affiliations:** 1 Department of Radiation Oncology, Key Laboratory of Cancer Prevention and Therapy, Tianjin’s Clinical Research Center for Cancer, Tianjin Medical University Cancer Institute & Hospital, National Clinical Research Center for Cancer, West Huanhu Road, West River District, Tianjin, 300060 China; 2 Department of Gastroenterology, Nanjing First Hospital, Nanjing Medical University, 300 Guangzhou Road, Nanjing, 210029 China; 3 Department of Gastrointestinal Oncology, Key Laboratory of Cancer Prevention and Therapy, Tianjin’s Clinical Research Center for Cancer, Tianjin Medical University Cancer Institute & Hospital, National Clinical Research Center for Cancer, West Huanhu Road, West River District, Tianjin, 300060 China

**Keywords:** Cervical cancer, Gut microbiota, 16S rRNA, Deep sequencing

## Abstract

Gut microbiota have been implicated in the development of many human diseases, including both digestive diseases and non-digestive diseases. In this study, we investigated whether the gut bacteria differed in cervical cancer (CCa) patients compared with healthy controls by 16S rRNA sequencing analysis. Subjects including eight CCa and five healthy controls were included. Microbiota profiles in fecal DNA were characterized by PCR amplification of the 16S rRNA V4 variable region and deep sequencing using the Illumina HiSeq platform. The CCa-associated gut microbiota had an increasing trend in alpha diversity, although statistical significance was not reached. Inter-group variability in community structure by beta diversity analysis showed a clear separation between cancer patients and healthy controls. Gut microbiota profiles were different between patients and controls; namely, the proportions of *Proteobacteria* phylum was notably higher in patients with CCa (ρ = 0.012). Seven genera differentiated significantly in relative abundance between CCa and controls (all ρ < 0.05), including *Escherichia*–*Shigella*, *Roseburia*, *Pseudomonas*, *Lachnoclostridium*, *Lachnospiraceae_UCG*-*004*, *Dorea* and *Succinivibrio*. The characteristic microbiome in CCa patients was also identified by linear discriminant analysis effect size (LEfSe). The phylum *Proteobacteria*, and the genus *Parabacteroides, Escherichia_Shigells* and *Roseburia* may provide novel potential biomarkers for CCa. Taken together, this is the first study on gut microbiota in patients with CCa, and demonstrated the significantly altered diversity and composition.

## Introduction

Cervical cancer is the fourth most common cause of cancer-related deaths in women worldwide, and the most common gynecological neoplasia in developing countries. Current literature reports that globally, there are approximately 500,000 new cases of cervical cancer, and more than 270,000 deaths annually (Basu et al. 2017). In recent decades, etiological factors such as infection with high-risk papilloma viruses (HPV) have been well established in cervical cancer. However, understanding of carcinogenesis is still insufficient, and the evidence suggests that many other host variations are important in the development of cervical cancer (Martin et al. 2007).

The human gastrointestinal tract carries about 10^14^ microbes. The genetic content of these microbial communities is approximately 100 times greater than seen in human genes (Human microbiome project consortium 2012). They co-exist with their hosts as a super-organism in a mutualistic manner and play fundamental roles in human health and disease. For example, emerging evidence shows that the intestinal microbiota regulates the host’s metabolism and also stimulates and renews epithelial cells. In addition, the intestinal microbiota will influence the development and maturation of the nervous and immune systems (Vrieze et al. 2013; Ursell et al. 2014; Dinan and Cryan 2017; Partida-Rodríguez et al. 2017). In return, individual signature contributes to differences in the gut microbiota. The structure of the gut microbial community changes constantly according to various external variables such as age, sex, stress, probiotic or antibiotic usage and genetic background (Sommer and Bäckhed 2013). Kozik et al. (2017) has demonstrated that the mouse fecal microbiome is partially shaped by factors such as sex, age and TNF production. These effects correlate with the severity of the animals’ colitis.

While several studies have investigated the role of the gut microbiota in the etiology of digestive disease: including inflammatory bowel disease (Pascal et al. 2017), hepatitis (Heidrich et al. 2018), and colorectal cancer (Wong et al. 2017), the effects on the development of cancer in other parts of the body have been limited. With the advent of next-generation 16S rRNA gene deep sequencing, the microbiome can now be characterized in a depth and detail that was not previously available. In this study, through 16S rRNA gene sequencing, we identified specific microbial signatures in patients with cervical cancer and sought to elucidate potential biomarkers or underlying mechanism how the microbiota may influence the pathogenesis of cervical cancer.

## Materials and methods

### Study participants

A total of eight patients with cervical cancer (CCa), who had not received any treatments were recruited in our department between June 2015 to January 2016. The detailed clinical parameters are shown in Table 1. Fecal samples were obtained 1 day after the patient was pathology confirmed. None of the patients had either used antibiotics or probiotics within 2 months or taken proton pump inhibitors within at least 2 weeks before sample collection. Exclusion criteria also included factors known to impact the intestinal microbiota, such as inflammatory bowel disease, existed abnormal bowel symptoms (e.g. abdominal pain, tenesmus, fecal incontinence or diarrhea), and other types of cancer (Pascal et al. 2017; Wong et al. 2017). Another five age-matched healthy female controls (HCs) were also enrolled. Detail informed written consent was obtained from all participants. The study was conducted according to the Declaration of Helsinki and approved by the Medical Ethics Committee of Tianjin Medical University Cancer Institute & Hospital.

**Table 1:** Patient characteristics

Characteristics	Values
Age (years)	59.5 (range 37–72)
Karnofsky performance score ≧ 70	8 (100%)
FIGO stage	
II	6 (75.0%)
III	2 (25.0%)
Histology	
Squamous carcinoma	7 (87.5%)
Adenocarcinoma	1 (12.5%)
Differentiation	
Well/moderate	5 (62.5%)
Poor	3 (37.5%)
Diameter of tumor (cm)	
≥ 4	3 (37.5%)
< 4	5 (62.5%)
Vaginal infiltration	
Presented	4 (50.0%)
None	4 (50.0%)
Lymph node metastasis	
Presented	5 (62.5%)
None	3 (37.5%)

### Sample collection and DNA extraction

Fecal samples were freshly collected by participants,. deposited in a sterile container containing RNAlater, and stored at − 20 °C. The QIAamp DNA Micro Kit (QIAGEN, Hilden, Germany) was used to extract microbial metagenomic DNA from 200 mg of each sample.

### 16S rRNA gene sequencing

PCR reactions were carried out using Phusion^®^ High-Fidelity PCR Master Mix (New England Biolabs). 16S rRNA genes of distinct V4 hypervariable region were amplified used bar-coded specific primer (515F-806R) as previously described prior to sequencing (Caporaso et al. 2011). After PCR products were purified, sequencing libraries were generated using TruSeq^®^ DNA PCR-Free Sample Preparation Kit (Illumina, USA), and then the index codes were attached. Quality of library was determined on the Qubit@ 2.0 Fluorometer (Thermo Scientific) and Agilent Bioanalyzer 2100 system followed by sequencing on the IlluminaHiSeq 2500 platform and generation of the paired-end reads (250 bp).

### Bioinformatics analysis

The paired-end reads obtained was assigned to samples based on barcode. Barcode and primer sequence were cut off and truncated reads were merged using FLASH (V1.2.7) (Magoč and Salzberg 2011). Quality filtering on the raw tags was performed with QIIME quality-controlled process (V1.7.0) as previously described (Caporaso et al. 2017). To detect chimera sequences, the clean tags were blasted with the reference database (http://drive5.com/uchime/uchime_download.html) using the UCHIME algorithm (http://www.drive5.com/usearch/manual/uchime_algo.html) (Edgar et al. 2011). The effective tags were obtained after remove of the chimera sequences (Haas et al. 2011).

Sequences analysis was carried out by Uparse software (V7.0.1001) (Edgar 2013). Sequences at 97% were assigned to the same operational taxonomic units (OTUs). In order to annotate taxonomic information, sequence for each OTU was screened using GreenGene Database (DeSantis et al. 2006) based on RDP classifier algorithm (V2.2) (Wang et al. 2007). At last, multiple sequence alignment was conducted using the MUSCLE software (V3.8.31), to analyze the phylogenetic relationship and the dominant species (Edgar 2004).

### Data analysis

Alpha diversity was calculated using Chao1 index, Shannon index, et al. with QIIME (V1.7.0) and displayed with R software (V2.15.3). Beta diversity was measured using both weighted and unweighted unifrac distance metrics by QIIME software (V1.7.0). Principal coordinate analysis (PCoA) was expressed by WGCNA package, ggplot2 packages and stat package in R software (V2.15.3). ρ < 0.05 was taken as statistical significance.

## Results

### Richness and diversity analysis

**Fig. 1:**
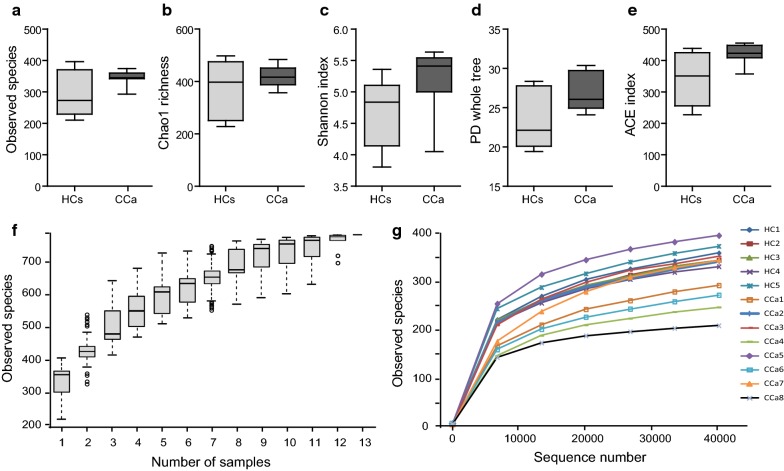
Analysis of alpha diversity in patient with cervical cancer compared with healthy controls. **a** Observed species, **b** Chao1 richness index, **c** Shannon index, **d** PD whole tree, **e** ACE index, **f** species accumulation curves, **g** rarefaction curves. Patient with cervical cancer (CCa), healthy controls (HCs)

In total, 783,660 reads, with an average of 64,361 reads per sample were generated after initial quality filtering (median read length = 253 bp). The total number of OTUs was 6665 at more than 97% similarity level. The alpha diversity indices, including observed species, Chao1, Shannon index, phylogenetic diversity (PD) whole tree, and abundance-based coverage estimator (ACE), were calculated for each data set (Table 2). Chao-1 is a measure of total richness and is particularly useful because of a valid variance which can be used to calculate confidence intervals (Chao 1987; Wang et al. 2005). The Shannon index reflects species numbers and evenness of species abundance (Guinane et al. 2013). PD whole tree reflects the sum of all branch-lengths on the constructed phylogenetic tree from all taxa (Goedert et al. 2015). Our results showed that the fecal microbiota of CCa patients had overall higher alpha diversity than those of the healthy controls, although no significant difference was observed by *t*-*test* (Fig. 1a–e).

**Table 2:** Estimation of diversity at the 97% similarity level within each data set

Sample	Shannon index	Simpson index	Chao1 richness	Good’s coverage
HC1	5.079	0.936	461.250	0.997
HC2	5.561	0.944	643.775	0.996
HC3	5.045	0.941	342.021	0.998
HC4	4.172	0.830	658.015	0.994
HC5	4.789	0.902	284.857	0.999
CCa1	6.023	0.966	552.016	0.996
CCa2	5.515	0.930	521.060	0.997
CCa3	5.912	0.962	547.294	0.997
CCa4	5.810	0.955	567.056	0.996
CCa5	5.999	0.961	516.623	0.997
CCa6	4.355	0.843	497.978	0.997
CCa7	5.123	0.888	530.344	0.997
CCa8	5.633	0.951	620.238	0.996

The species accumulation curve reached asymptotic values in each sample, revealing that the sampling effort was sufficient to figure out the most genera present (Fig. 1f). The rarefaction curve reached a summit in each sample, indicating that the sequencing depth was sufficient to detect all the genera within each sample and beneficial to capture the microbial diversity (Fig. 1g).

**Fig. 2:**
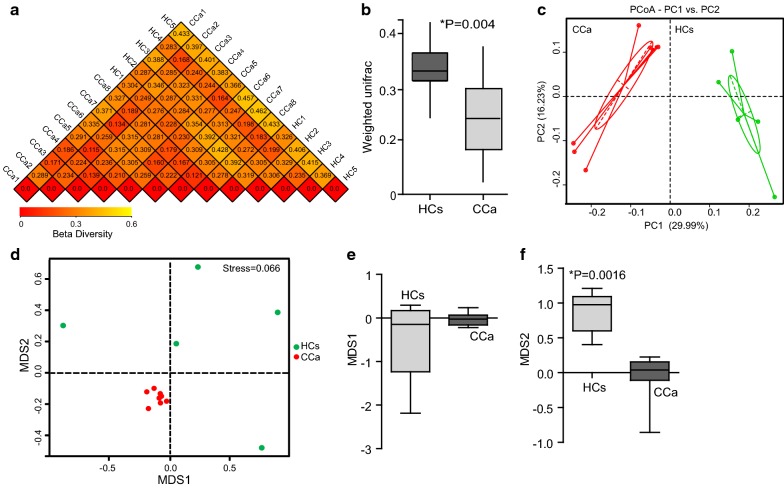
Analysis of beta diversity in patient with cervical cancer compared with healthy controls. **a** Heatmap and **b** histogram of beta diversity based of Unifrac distance matrices (P = 0.004 by *t*-*test*). **c** PCoA analysis based of Unifrac distance matrices. Each sphere represents one sample. Samples separate into two clusters. **d** NMDS plots. (*Green* healthy control samples, HC1-HC5; *Red* patient samples, CCa1–CCa8). **e, f** Differences across groups are established at the first and second MDS (**e**, MDS1 and **f**, MDS2) values. MDS1, P > 0.05; MDS2, P = 0.0016 by Mann–Whitney U test

Beta diversity was first evaluated with weighted-UniFrac analysis (Fig. 2a, b, ρ = 0.004). UniFrac-based PCoA provided an entire comparison of microbial communities, and showed that the CCa cohort and the HC cohort show clear separation (Fig. 2c). Furthermore, non-metric multi-dimensional scaling (NMDS) showed that the fecal microbiota of CCa group was distinct from the HC group (Fig. 2d ANOSIM, R = 0.6224, ρ = 0.001; MRPP, A = 0.07559, observed-delta = 0.5686, expected-delta = 0.6151, ρ = 0.004). NMDS analysis based on Sorensen (Bray–Curtis) distance indicated noted differences in microbial communities at the second MDS between CCa group and HC group (MDS1, ρ > 0.05; MDS2, ρ = 0.0016; Mann–Whitney U test, Fig. 2e, f).

### Comparative analysis of the gut microbial composition of patients with cervical cancer and healthy controls

**Fig. 3:**
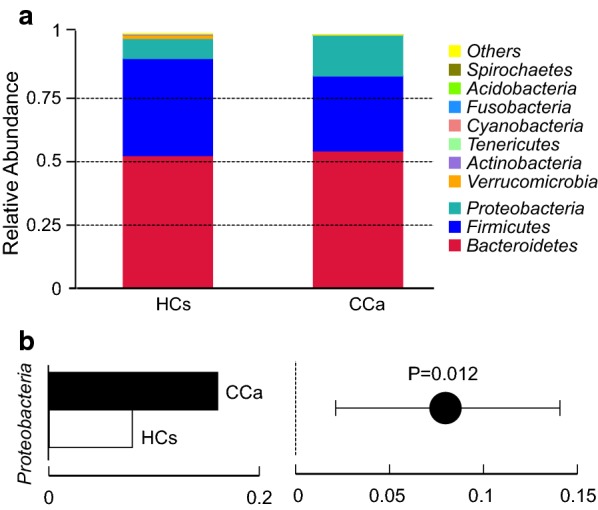
Composition of gut microbiota in patient with cervical cancer on the phylum level. **a** The relative abundance of bacteria in the stool sample from the two groups. **b** The relative abundance of *Proteobacteria* was significantly higher in patients compared with controls (P = 0.012)

Assigned sequence reads were used to assess differences in taxonomic abundances between CCa and HCs at various levels. At the phylum level, *Bacteroidetes* was the most abundant, contributing 54.19% and 51.96% of the gut microbiota in CCa group and HC group respectively, followed by *Firmicutes* (29.55% and 16.00% respectively) and *Proteobacteria* (38.28% and 7.9% respectively) (Fig. 3a). Microbial compositions showed high inter-individual variability. For example, *Bacteroidetes* accounted for 34.08–68.82%, *Firmicutes* 18.57–47.24%, and *Proteobacteria* 3.79–25.95% among all the individuals. The relative abundance of *Proteobacteria* in CCa patients was significantly higher (ρ = 0.012), while over-abundance of *Bacteroidetes* and *Firmicutes* did not reach statistical significance (Fig. 3b).

**Fig. 4:**
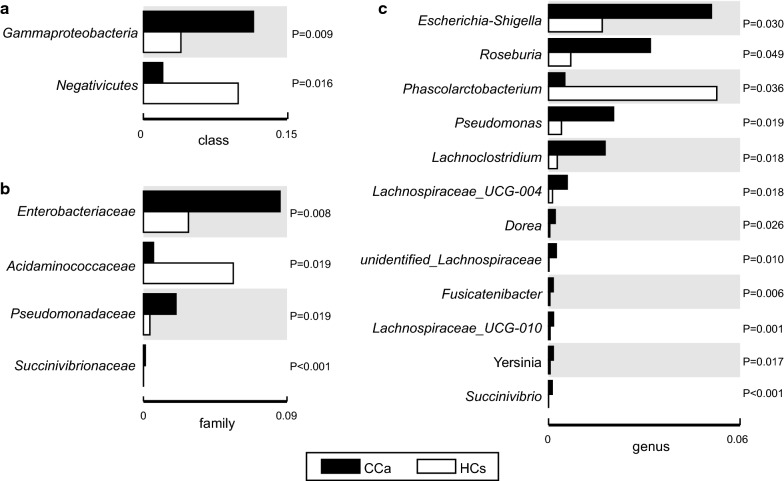
Boxplots representing the average proportion of each 16S sequence read attributed to each taxon. **a** on the class level, **b** on the family level, **c** on the genus level (*White* healthy control samples, *Black* patient samples**)**

At the class level, a significant increase was observed in the abundance of *Gammaproteobacteria* in CCa group (Fig. 4a, ρ = 0.009). Moreover, upon closer examination of taxonomic data, we noted that CCa group were enriched with order *Enterobacteriales* (ρ = 0.008), *Aeromonadales* (ρ < 0.001), *Oceanospirillales* (ρ = 0.020) and *Alteromonadales* (ρ = 0.001) from the *Gammaproteobacteria* class (Fig. 4b).

At the family level, eight families were presented at significantly altered proportions (ρ < 0.05) in CCa patients compared to HCs. Five families increased including *Enterobacteriaceae* (ρ = 0.008), *Pseudomonadaceae* (ρ = 0.019), *Succinivibrionaceae* (ρ < 0.001) and *Halomonadaceae* (ρ = 0.008). Whereas *Acidaminococcaceae* were decreased (ρ = 0.019).

Genus-level analysis was more informative (Fig. 4c and Table 3). The data revealed that genus *Phascolarctobacterium* (ρ = 0.036) was notably overabundant in HCs, possibly inflating the high *Firmicutes* abundance at the phylum level. While genera *Escherichia*–*Shigella* (ρ = 0.030) and *Roseburia* (ρ = 0.049) were more abundant in CCa group. Other minor genera that were also significantly enriched in CCa patients were *Pseudomonas* (ρ = 0.018), *Lachnoclostridium* (ρ = 0.018), *Lachnospiraceae_ UCG*-*004* (ρ = 0.018), *Dorea* (ρ = 0.026), and *Succinivibrio* (ρ < 0.001).

**Table 3:** Genera differences between patients and controls

Genus	HC (mean)	CCa (mean)	P value*
Higher in HC group			
*Phascolarctobacterium*	0.052614	0.005016	0.036
*Halomonas*	0.000244	0.000932	0.003
Higher in CCa group			
*Succinivibrio*	0.0066 × 10^−3^	0.001134	0.000
*Ruminococcus*	0.000422	0.007318	0.019
*Morganella*	0.00198 × 10^−2^	0.000507	0.019
*Shewanella*	0.0066 × 10^−2^	0.000285	0.002
*Proteus*	0.0066 × 10^−3^	0.000132	0.005
*Dorea*	0.000323	0.001902	0.006

*By non-parametric *t*-*test*

### Comparative analysis of the gut microbial taxa between patients with cervical cancer and healthy controls

We further compared taxa in the CCa vs. HC groups by discriminant analysis effect size (LEfSe). The LEfSe method is used to discover high-dimensional biomarker. The linear discriminant analysis (LDA) model identifies differently abundant taxa between groups and estimates the effect size of each significantly different taxon (Segata et al. 2011).

**Fig. 5:**
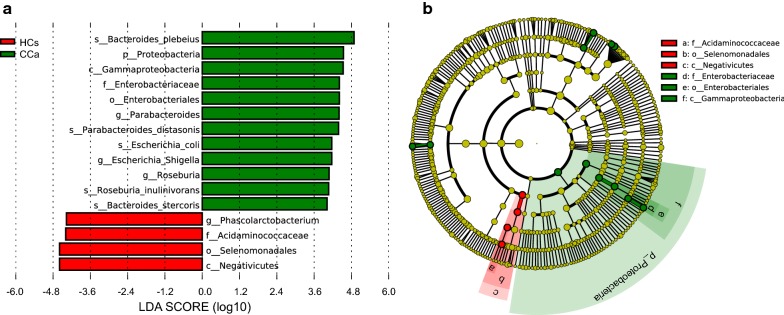
Taxonomic differences were detected between cervical cancer and healthy controls. **a** Linear discriminative analysis (LDA) effect size (LEfSe) analysis between healthy controls (HCs, *red*) and patients (CCa, *green*). **b** Cladogram showing differentially abundant taxonomic clades with an LDA score > 4.0 among patients and controls

LEfSe analysis revealed that the phylum *Proteobacteria*, and the genus *Parabacteroides, Escherichia_Shigells,* and *Roseburia* were all significantly more abundant in the fecal samples from the patients with CCa and conversely, significantly less abundant in the fecal samples from healthy controls (Fig. 5a).

A cladogram shown in Fig. 5b represented the connection between the significantly different taxa at different taxonomic levels. For example, *Enterobacteriaceae* (family) is under *Enterobacteriales* (order) which is under *Gammaproteobacteria* (class). A clade is a branch of organisms under a common ancestor (e.g. *Proteobacteria* is a common ancestor for all the genera belong to it). The significantly different taxa was shown in a tree like structure. Moreover, LDA score demonstrated that these differentially abundant taxa can be considered as potential biomarkers (LDA score > 4.0, ρ < 0.05).

## Discussion

Cervical cancer is a heterogeneous and multifactorial disease, impacted by several different genetic and environmental factors. Although infection with HPV as a prerequisite for cervical cancer has been conclusively proven, the specific impact of other factors on this disease process is not yet well-characterized. In this study, we identified for the first time the significant alterations in gut microbial composition following cervical cancer development and evaluated the role of gut microbiota in the pathogenesis of cervical cancer. We showed that (i) there was a trend towards increased diversity within samples in the cervical cancer patients, and an obvious situation that gut microbiota community segregated between the CCa group and HC group, (ii) the gut microbial composition differs significantly in patients with cervical cancer compared to healthy subjects, and (iii) *Bacteroidetes* were the most predominant abundant bacterial taxa in the cervical cancer fecal specimens, while *Firmicutes* presented a relatively strikingly low abundance. Further LEfSe analysis indicated that gut microbiota taxa present could be used to differentiate cervical cancer patients from controls and thus could be regarded as potential biomarkers of clinical relevance. Taken together, this study demonstrates that patients with cervical cancer have their unique characteristic gut microbiota.

The connection of gut microbiota and both digestive diseases (Pascal et al. 2017; Wong et al. 2017; Heidrich et al. 2018) and non-digestive diseases (Scher and Abramson 2011; Dinan and Cryan 2017; Jie et al. 2017) have been suggested by numerous studies. However, most previously published studies were based on low throughput methods, such as traditional microbial culture, real-time fluorescent quantitative PCR and 16S rRNA amplicon denaturing gradient gel electrophoresis. The results revealed only a small fraction of the overall microorganisms. Therefore, the overall picture of intestinal microbial communities and the corresponding microbial ecology are far from well understood. Over the past few years, the development and increased availability of next generation sequencing technologies, including high-throughput 16S rRNA gene sequencing, has facilitated our understanding, and enabled astonishing discoveries about the microbial gene repertoire. Bacterial 16S rRNA genes generally contain nine “hypervariable regions” (V1–V9) which represent considerable sequence diversity among different bacteria. Studies have isolated those sequences that identify a single bacterial species or differentiate between a limited number of different species (Stohr et al. 2005; Chakravorty et al. 2007). In this study, we examined bacterial taxonomic composition and phylogenetic diversity by PCR amplification of the 16S rRNA V4 variable region and deep sequencing on the Illumina^®^ HiSeq platform. The results were in consistent with other microbiome studies. Altered microbial diversity was observed in the fecal communities of cervical cancer patients, and they have overabundance of the genera indicated. On the other hand, data from different studies are scattered as proposed by Li et al. (2014). There is no comprehensive and uniformly processed database that represents the human gut microbiota worldwide; and moreover, it is also not clear at what pace the number of species and genes will continue to grow, with the increasing amount of sequencing data.

Cervical lesions are always linked to abnormal vaginal microbiota. Persistent infection with high-risk HPV is directly involved in the tumorigenesis of approximately 70% cases of cervical cancer (Ramakrishnan et al. 2015). HPV-positive women had vaginal microbiomes with greater bacterial diversity, including specifically being abundant in *L. gasseri* and *G. vaginalis* (Gao et al. 2013). Greater number of *L. gasseri* was associated with rapid remission of HPV (Brotman et al. 2014), and therefore a low risk of developing HPV-associated malignant transformation. However there are few studies of gut microbiota and cervical cancer. This study is, to our knowledge, the first time that the gut microbiota of cervical cancer patients have been analyzed by comprehensive next-generation sequencing, independent of culture methods. Patients with cervical cancer presented with a distinct composition of gut microbiota compared to healthy subjects, and the gut microbial communities may play a role in promoting cervical tumorigenesis. However, the exact mechanism is unclear. Firstly, bacterial microbiome-induced tumorigenesis is thought to be associated with inflammatory response mediated by microorganism-associated molecular patterns (MAMP) and their activation of pattern recognition receptors (PRRs). Upon a MAMP binding to a PRR (such as Toll-like receptor), transcription of antibacterial proteins was stimulated by intracellular signaling cascade in the host epithelial cell. And pro-inflammatory cytokines including IL-17, TNF-α, and IFN-γ was also upregulated (Cerf-Bensussan and Gaboriau-Routhiau 2010). This inflammatory response occurs not only locally, but more importantly at a systemic level, and thereby increases the risk of inflammation at distant sites (van der Meulen et al. 2016). Besides, gut microbiota modulate the enterohepatic circulation of estrogens, which circulate to exert effects on target organs like breast and uterine cervix (Goedert et al. 2015; van der Meulen et al. 2016). Chung’s mouse studies have provided strong evidence that estrogen contributes to cervical carcinogenesis (Chung et al. 2010). Epidemiological data also indicated that women with the highest levels of circulating estrogen are at increased risk of developing cervical cancer (Chung et al. 2010). Otherwise, the gut microbial differences might affect cervical cancer risk through many other pathways, and further biofunctional studies are needed.

There are some limitations of this study. The number of enrolled patients in each subgroup was relatively small. Eight patients and five controls were collected, and thus we are unable to definitively distinguish between different stages of cancer and precancerous changes. Secondly, the risk factors, such as persistent positive HPV and many sexual partners were not measured. The association of risk factors and gut bacteria was not analyzed as well. Moreover, functional roles of identified bacterial species and interrelation to tumorigenesis of cervical cancer remain unclear.

In conclusion, we reported the comprehensive analysis of gut microbiota in patients with cervical cancer using a relatively small stool samples. Such analysis for diagnosis, prediction the risk of recurrence, and prevention using probiotics or antibiotics should be assessed in the future.

## Abbreviations

ACE: abundance-based coverage estimator
CCa: cervical cancer
HC: healthy control
LDA: linear discriminant analysis
LEfSe: discriminant analysis effect size
OTUs: operational taxonomic units
PCoA: principal coordinate analysis
PD: phylogenetic diversity
